# Supplementary material for: Neonicotinoid Microsphere Immunosensing for Profiling Applications in Honeybees and Bee-Related Matrices
Source: Biosensors (Basel). 2022 Sep 26;12(10):792. doi: 10.3390/bios12100792 (PMC9599487; doi:10.3390/bios12100792)
Supplement: Supplementary file 1 [file biosensors-12-00792-s001.zip › biosensors-1865416-Supplementary Materials.pdf]

Supporting Information

Tables

Table S1. Details for all neonicotinoid microsphere immunoassays (nMIAs).

Name	Microsphere	Main target	Hapten	Antibody
Imi-assay	MC10066	Imidacloprid	Imidacloprid-OVA (Imi-OVA)	Imidacloprid mAb (Imi mAb)
Ace-Assay	MC10064	Acetamiprid	Acetamiprid-OVA (Ace-OVA)	Acetamiprid mAb (Ace mAb)
Clo-Assay	MC10012	Clothianidin	Clothianidin-OVA (Thc-OVA)	Clothianidin mAb (Clo mAb)
Thc-Assay	MC10038	Thiacloprid	Thiacloprid-OVA (Thc-OVA)	Thiacloprid mAb (Thc mAb)
Thm Assay	MC10052	Thiamethoxam	Thiamethoxam-OVA(Thm-OVA)	Thiamethoxam mAb (Thm mAb)

Table S2. Collected blank water samples.

Sample No.	Country of Origin	Sample Location	Sampling Date
1	the Netherlands	Oosterbeek, brook	2019/12/30
2	the Netherlands	Saphatipark pond, Amsterdam	2019/12/30
3	the Netherlands	Leuvehaven harbour, Rotterdam	2019/12/28
4	the Netherlands	Stokviswater canal, Rotterdam	2019/12/28
5	Belgium	Minnewater city lake, Bruges	2019/12/30
6	Peru	Aquaduct, Lima	2019/12/27
7	the Netherlands	Rhine River, Oosterbeek	2019/12/28
8	Belgium	Botanic garden pond, Brussel	2019/12/30
9	the Netherlands	Tapwater, Wageningen	2019/12/18
10	France	Seine River, Paris	2019/12/23

Table S3. Detection characteristics of the five nMIAs based on four-parameter logistic analysis of the dose-response curves (n=2).

Assay	LOD (ng/mL)[1]	IC_{50} (ng/mL)[2]	Dynamic Range (ng/mL)[3]	(R^2)[4]
Imidacloprid	0.01	0.07	0.02-0.17	0.9997
Acetamiprid	0.02	0.26	0.04-6.10	0.9982
Clothianidin	0.19	1.9	0.51-10	0.9992
Thiacloprid	0.02	0.06	0.02-1.23	0.9981
Thiamethoxam	0.003	0.01	0.001-0.63	0.9987

[1] Based on average background value of the negative control minus three times the standard deviation, [2] 50% of the maximal inhibitory concentration, [3] dynamic measurement range, set between IC_{80} to IC_{20}, [4] Coefficient of determination.

Table S4. Overview of best neonicotinoid profiling sensitivities based on fortification in surface water by the five nMIAs.

Neonicotinoid	Imi Assay	Ace Assay	Clo Assay	Thc Assay	Thm Assay
Imidacloprid	1 ng/mL	-	-	10 ng/mL	-
Acetamiprid	1 ng/mL	1 ng/mL	-	1 ng/mL	-
Clothianidin	1 ng/mL	-	10 ng/mL	10 ng/mL	-
Thiacloprid	1 ng/mL	1 ng/mL	10 ng/mL	1 ng/mL	-
Thiamethoxam	-	-	-	1 ng/mL	1 ng/mL
Dinotefuran	-	-	1 ng/mL	-	-
Nitenpyram	1 ng/mL	-	-	-	-
Imidaclothiz	1 ng/mL	-	-	1 ng/mL	-

"-" means undetectable with the particular assay.

Figures

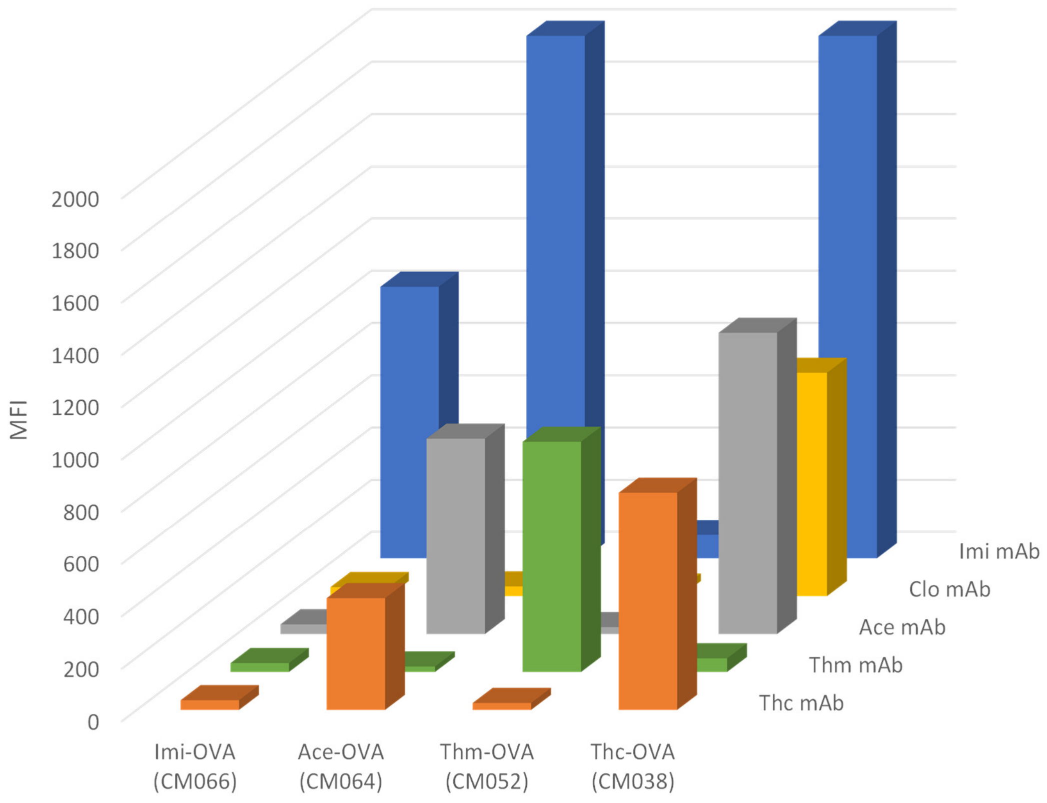

Figure S1. Cross-interactions between the neonicotinoid mAbs and the neonicotinoid-OVA conjugates on the microspheres.

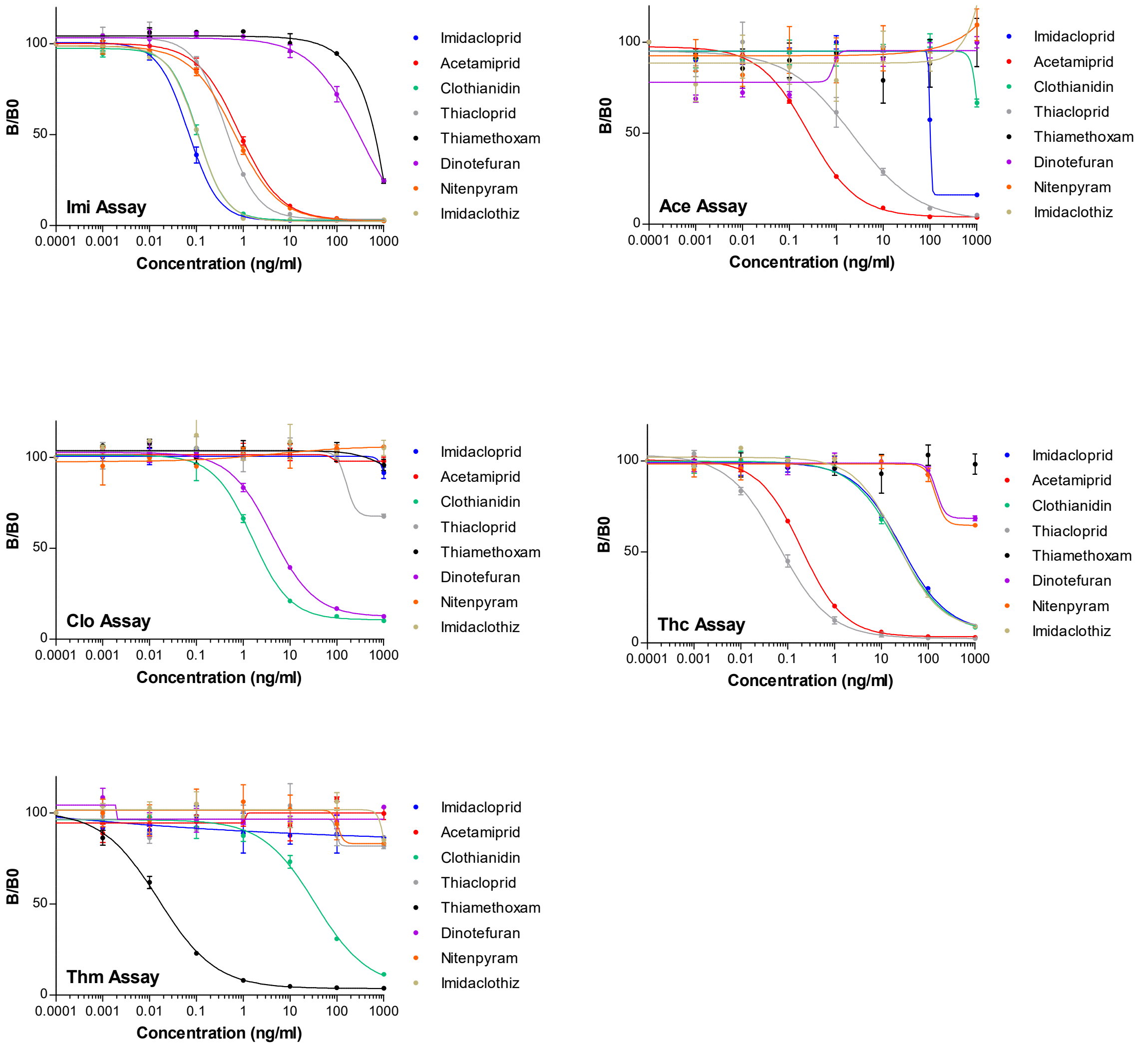

Figure S2. Dose-response curves-based cross-reactivity testing for all eight neonicotinoids in the five nMIAs.

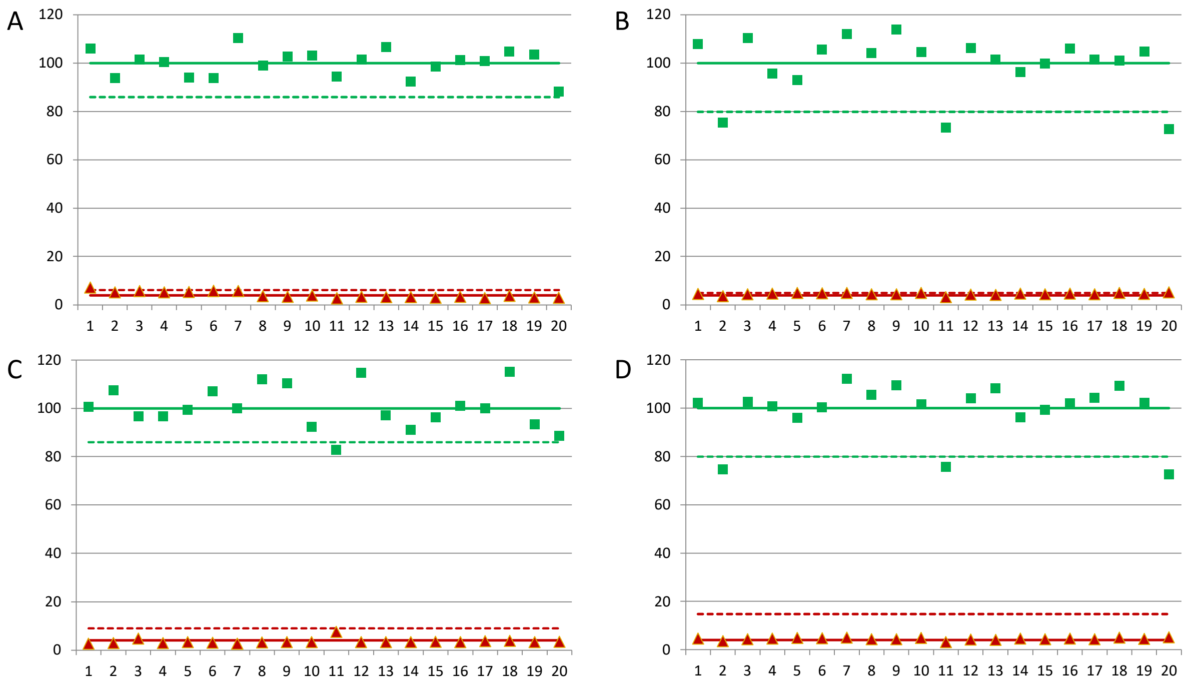

Figure S3. Graphical display of the validation results for the Imi (A), Clo (B), Thc (C) and Thm (D) assays for screening application in surface waters. The blank water samples were fortified with 5 ng/mL imidacloprid, clothianidin, thiacloprid and thiamethoxam, and measured with the corresponding nMIA for three consecutive days. Each triangle/square indicates an independent measurement of one sample in triplicate (n=3). The fortified samples are indicated by the red triangles and their mean value is indicated by the red solid lines. The blank samples are indicated by the green squares and their mean value is indicated by the green solid lines. The red dotted lines indicate the cut-off factor (Fm) and the green dotted line indicates the threshold level (T).

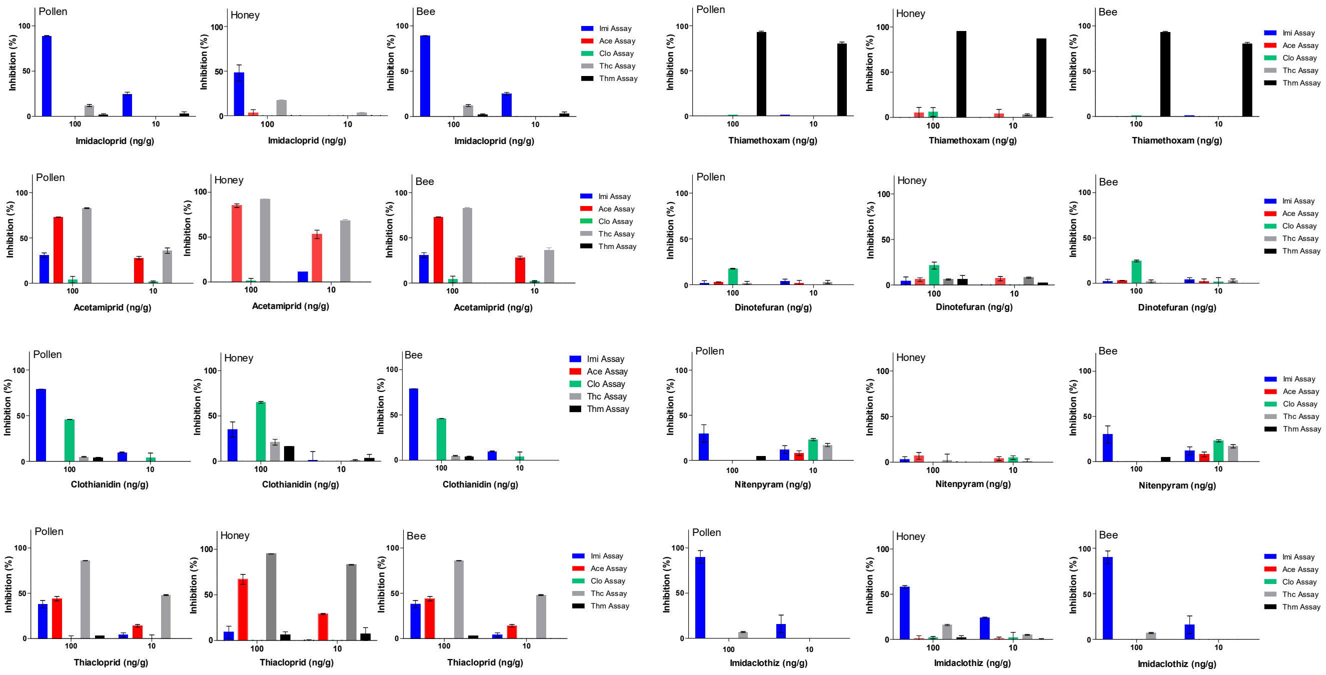

Figure S4. Neonicotinoid profiling patterns based on inhibition percentages of the eight common neonicotinoids at 100 and 10 ng/mL in the developed five nMIAs applied to bee-related matrices (pollen, honey and bees).
